# 4-(4-Methoxy­phen­yl)-7,7-dimethyl-5-oxo-5,6,7,8-tetra­hydrochromene-2,5-dione

**DOI:** 10.1107/S160053680904971X

**Published:** 2009-11-25

**Authors:** Hao Shi

**Affiliations:** a The College of Pharmaceutical Science, Zhejiang University of Technology, Hangzhou 310014, People’s Republic of China

## Abstract

The title compound, C_18_H_20_O_4_, was synthesized by the reaction of 4-methoxy­benzaldehyde, 2,2-dimethyl-1,3-dioxane-4,6-dione and 5,5-dimethyl­cyclo­hexane-1,3-dione with triethyl­benzyl­ammonium chloride in water as a green solvent. In the mol­ecule of the title compound, the six-membered pyran­one ring of the hexa­hydro­coumarin system has a screw-boat conformation while that of the dimethyl­cyclo­hexenone system has a distorted envelope conformation. The CMe_2_ portion of this ring is disordered over two positions with refined occupancies of 0.721 (7) and 0.279 (7).

## Related literature

For background to the applications of coumarin derivatives see: Wang *et al.* (1999 ▶); Yang (2001 ▶). For ring puckering parameters, see: Cremer & Pople (1975 ▶).
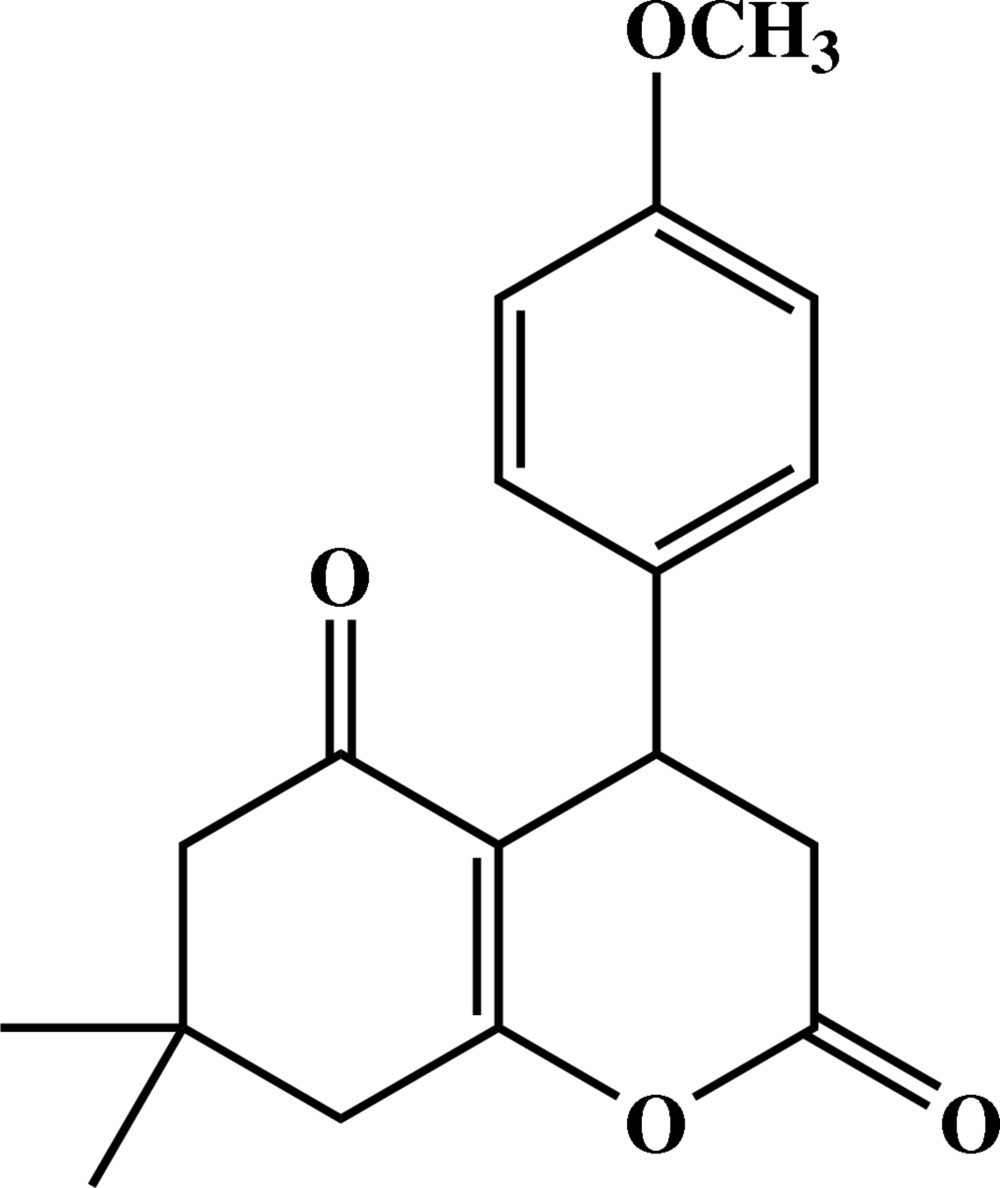


## Experimental

### 

#### Crystal data

C_18_H_20_O_4_
*M* *_r_* = 300.34
Orthorhombic, 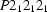

*a* = 5.9793 (6) Å
*b* = 11.7371 (12) Å
*c* = 22.565 (2) Å
*V* = 1583.6 (3) Å^3^
*Z* = 4
Mo *K*α radiation
μ = 0.09 mm^−1^
*T* = 298 K
0.37 × 0.21 × 0.17 mm

#### Data collection

Bruker SMART CCD area-detector diffractometer
Absorption correction: multi-scan (*SADABS*; Bruker, 1999 ▶) *T*_min_ = 0.968, *T*_max_ = 0.985
7934 measured reflections
1643 independent reflections
1288 reflections with *I* > 2σ(*I*)
*R*_int_ = 0.075

#### Refinement

*R*[*F* ^2^ > 2σ(*F* ^2^)] = 0.046
*wR*(*F* ^2^) = 0.128
*S* = 1.05
1643 reflections
233 parameters
H-atom parameters constrained
Δρ_max_ = 0.28 e Å^−3^
Δρ_min_ = −0.26 e Å^−3^

### 

Data collection: *SMART* (Bruker, 1999 ▶); cell refinement: *SAINT* (Bruker, 1999 ▶); data reduction: *SAINT*; program(s) used to solve structure: *SHELXS97* (Sheldrick, 2008 ▶); program(s) used to refine structure: *SHELXL97* (Sheldrick, 2008 ▶); molecular graphics: *ORTEP-3* (Farrugia, 1997 ▶); software used to prepare material for publication: *SHELXL97*.

## Supplementary Material

Crystal structure: contains datablocks I, global. DOI: 10.1107/S160053680904971X/sj2689sup1.cif

Structure factors: contains datablocks I. DOI: 10.1107/S160053680904971X/sj2689Isup2.hkl

Additional supplementary materials:  crystallographic information; 3D view; checkCIF report

## Figures and Tables

**Fig. 1.**
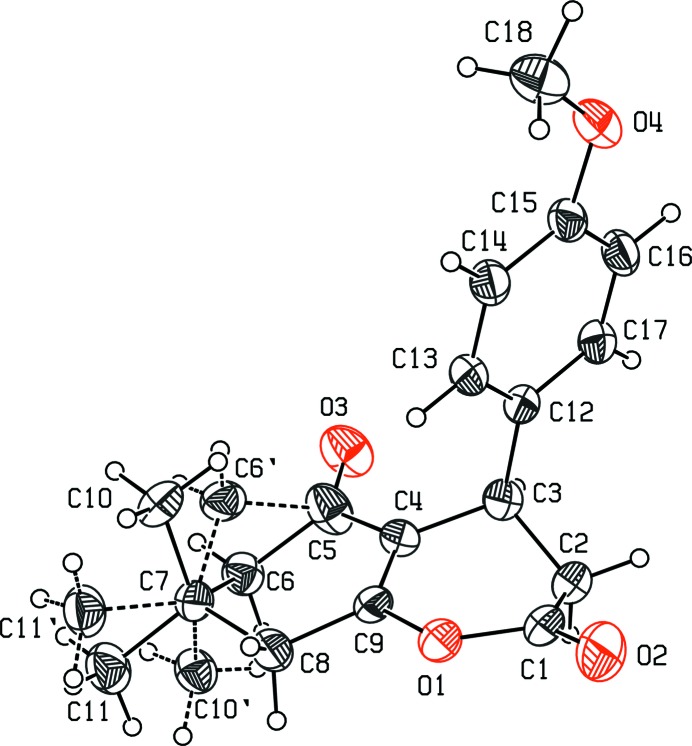
Structure of 1 showing 30% probability displacement ellipsoids and the atom numbering scheme. Bonds to atoms of the minor disorder component are shown as dashed lines.

**Fig. 2.**
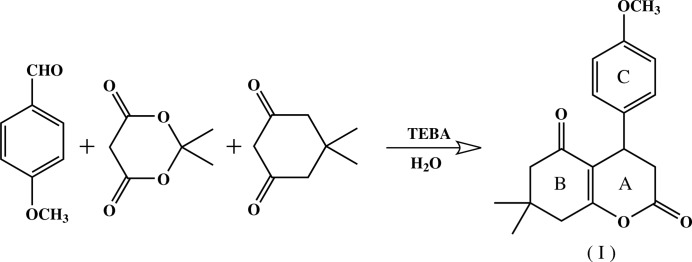
The preparation of the title compound.

## supplementary crystallographic information

### Comment

Coumarin is an important chemical with unique characteristics. It is widely used in hand soaps, detergents, lotions and laser dyes (Wang *et al.*, 1999). Coumarin and some of its derivatives have been tested in pharmacology for treatment of HIV (Yang, 2001). To obtain a coumarin in a more environmentally friendly way, water was used as a green solvent in the synthesis of the title compound.

In the molecule of the title compound, the two six membered rings of the hexahydrocoumarin system are not planar, the cyclohexene ring A (O1/C1–C4/C9) adopts the screw-boat conformation with puckering parameters (Cremer & Pople, 1975) Q= 0.468 (5) Å, θ= 64.4 (5)° and φ = 143.5 (6)°; for the ring B (C4–C9), disorder was modelled for the C6, C10, C11 atoms resolved over two positions with occupancies of 0.721 (7) and 0.279 (7). Ring C (C12–C17) is, of course, planar. The dihedral angle between the least-squares plane of ring A(O1/C1–C4/C9) and that of ring C (C12–C17) is 87.59 (12)°.

### Experimental

A mixture of 4-methoxybenzaldehyde (100 mmol), 5,5-dimethyl-1,3-cyclohexanedione (100 mmol), 2,2-dimethyl-1,3-dioxane-4,6-dione (100 mmol), triethylbenzylammonium chloride(TEBA) (15 mmol) and 400 mL of water was stirred at 65°C for 4 h (Fig.2). The reaction mixture was cooled to room temperature, the precipitated product was filtered and recrystallized from ethanol to give the title compound. Crystals suitable for X-ray structure analysis were obtained by slow evaporation from methanol solution at room temperature.

### Refinement

All H atoms were placed in calculated positions and constrained to ride on their parent atoms with C–H distances in the range 0.93–0.98 Å, They were treated as riding atoms, with *U*_iso_(H) = 1.5Ueq(C) for the methyl H atoms and 1.2Ueq(C) for other H atoms. In the absence of significant anomalous scattering, the absolute configuration could not be reliably determined from the X-ray data and Friedel pairs were merged.

### Crystal data

**Table:**

C_18_H_20_O_4_	*F*(000) = 640
*M**_r_* = 300.34	*D*_x_ = 1.260 Mg m^−^^3^
Orthorhombic, *P*2_1_2_1_2_1_	Mo *K*α radiation, λ = 0.71073 Å
Hall symbol: P 2ac 2ab	Cell parameters from 2932 reflections
*a* = 5.9793 (6) Å	θ = 2.5–25.3°
*b* = 11.7371 (12) Å	µ = 0.09 mm^−^^1^
*c* = 22.565 (2) Å	*T* = 298 K
*V* = 1583.6 (3) Å^3^	Prism, colorless
*Z* = 4	0.37 × 0.21 × 0.17 mm

### Data collection

**Table:**

Bruker SMART CCD area-detector diffractometer	1643 independent reflections
Radiation source: fine-focus sealed tube	1288 reflections with *I* > 2σ(*I*)
graphite	*R*_int_ = 0.075
φ and ω scans	θ_max_ = 25.0°, θ_min_ = 1.8°
Absorption correction: multi-scan (*SADABS*; Bruker, 1999)	*h* = −7→6
*T*_min_ = 0.968, *T*_max_ = 0.985	*k* = −13→10
7934 measured reflections	*l* = −25→26

### Refinement

**Table:**

Refinement on *F*^2^	Secondary atom site location: difference Fourier map
Least-squares matrix: full	Hydrogen site location: inferred from neighbouring sites
*R*[*F*^2^ > 2σ(*F*^2^)] = 0.046	H-atom parameters constrained
*wR*(*F*^2^) = 0.128	*w* = 1/[σ^2^(*F*_o_^2^) + (0.0461*P*)^2^ + 0.553*P*] where *P* = (*F*_o_^2^ + 2*F*_c_^2^)/3
*S* = 1.05	(Δ/σ)_max_ = 0.001
1643 reflections	Δρ_max_ = 0.28 e Å^−^^3^
233 parameters	Δρ_min_ = −0.26 e Å^−^^3^
0 restraints	Extinction correction: *SHELXL97* (Sheldrick, 2008), Fc^*^=kFc[1+0.001xFc^2^λ^3^/sin(2θ)]^-1/4^
Primary atom site location: structure-invariant direct methods	Extinction coefficient: 0.031 (4)

### Special details

**Table:**

Geometry. All e.s.d.'s (except the e.s.d. in the dihedral angle between two l.s. planes) are estimated using the full covariance matrix. The cell e.s.d.'s are taken into account individually in the estimation of e.s.d.'s in distances, angles and torsion angles; correlations between e.s.d.'s in cell parameters are only used when they are defined by crystal symmetry. An approximate (isotropic) treatment of cell e.s.d.'s is used for estimating e.s.d.'s involving l.s. planes.
Refinement. Refinement of *F*^2^ against ALL reflections. The weighted *R*-factor *wR* and goodness of fit *S* are based on *F*^2^, conventional *R*-factors *R* are based on *F*, with *F* set to zero for negative *F*^2^. The threshold expression of *F*^2^ > σ(*F*^2^) is used only for calculating *R*-factors(gt) *etc*. and is not relevant to the choice of reflections for refinement. *R*-factors based on *F*^2^ are statistically about twice as large as those based on *F*, and *R*- factors based on ALL data will be even larger.

### Fractional atomic coordinates and isotropic or equivalent isotropic displacement parameters (Å^2^)

**Table:**

	*x*	*y*	*z*	*U*_iso_*/*U*_eq_	Occ. (<1)
O1	0.2602 (5)	0.1771 (2)	0.05280 (10)	0.0674 (8)	
O2	0.1786 (9)	0.0959 (3)	−0.03152 (13)	0.1292 (18)	
O3	0.8756 (6)	0.0717 (3)	0.17213 (14)	0.0865 (10)	
O4	0.2049 (6)	−0.4043 (2)	0.11499 (12)	0.0742 (9)	
C1	0.3211 (11)	0.1093 (3)	0.00511 (16)	0.0782 (15)	
C2	0.5491 (10)	0.0631 (4)	0.00509 (16)	0.0766 (15)	
H2A	0.6523	0.1218	−0.0079	0.092*	
H2B	0.5577	0.0005	−0.0229	0.092*	
C3	0.6193 (8)	0.0208 (3)	0.06653 (15)	0.0596 (10)	
H3	0.7809	0.0069	0.0657	0.072*	
C4	0.5755 (7)	0.1163 (3)	0.10917 (15)	0.0498 (9)	
C5	0.7227 (8)	0.1351 (4)	0.1596 (2)	0.0757 (13)	
C6	0.7088 (10)	0.2528 (4)	0.1872 (2)	0.0597 (17)	0.721 (7)
H6A	0.7831	0.3072	0.1616	0.072*	0.721 (7)
H6B	0.7866	0.2523	0.2249	0.072*	0.721 (7)
C6'	0.584 (3)	0.1803 (12)	0.2150 (6)	0.064 (5)	0.279 (7)
H6'1	0.4749	0.1236	0.2271	0.077*	0.279 (7)
H6'2	0.6836	0.1945	0.2481	0.077*	0.279 (7)
C7	0.4652 (6)	0.2907 (3)	0.19705 (14)	0.0516 (9)	
C8	0.3415 (7)	0.2858 (3)	0.13786 (14)	0.0591 (10)	
H8A	0.1821	0.2814	0.1455	0.071*	
H8B	0.3695	0.3559	0.1163	0.071*	
C9	0.4070 (7)	0.1883 (3)	0.10012 (13)	0.0502 (9)	
C10	0.3528 (11)	0.2039 (5)	0.2381 (2)	0.0695 (19)	0.721 (7)
H10A	0.4307	0.2017	0.2753	0.104*	0.721 (7)
H10B	0.3571	0.1299	0.2201	0.104*	0.721 (7)
H10C	0.2001	0.2258	0.2447	0.104*	0.721 (7)
C11	0.4615 (15)	0.4084 (6)	0.2232 (3)	0.080 (2)	0.721 (7)
H11A	0.5441	0.4088	0.2597	0.119*	0.721 (7)
H11B	0.3097	0.4307	0.2308	0.119*	0.721 (7)
H11C	0.5286	0.4611	0.1959	0.119*	0.721 (7)
C10'	0.651 (3)	0.3774 (11)	0.1822 (7)	0.062 (4)	0.279 (7)
H10D	0.5839	0.4465	0.1678	0.094*	0.279 (7)
H10E	0.7469	0.3463	0.1522	0.094*	0.279 (7)
H10F	0.7361	0.3934	0.2172	0.094*	0.279 (7)
C11'	0.326 (4)	0.3516 (15)	0.2465 (7)	0.073 (5)	0.279 (7)
H11D	0.4237	0.3733	0.2783	0.109*	0.279 (7)
H11E	0.2137	0.3005	0.2612	0.109*	0.279 (7)
H11F	0.2560	0.4182	0.2303	0.109*	0.279 (7)
C12	0.5050 (7)	−0.0900 (3)	0.08200 (13)	0.0496 (9)	
C13	0.3053 (7)	−0.0949 (3)	0.11313 (14)	0.0522 (9)	
H13	0.2407	−0.0275	0.1263	0.063*	
C14	0.1992 (7)	−0.1971 (3)	0.12516 (14)	0.0545 (9)	
H14	0.0657	−0.1980	0.1463	0.065*	
C15	0.2929 (7)	−0.2976 (3)	0.10557 (15)	0.0533 (9)	
C16	0.4910 (7)	−0.2948 (3)	0.07371 (15)	0.0595 (10)	
H16	0.5539	−0.3623	0.0601	0.071*	
C17	0.5949 (7)	−0.1928 (3)	0.06213 (14)	0.0570 (9)	
H17	0.7277	−0.1922	0.0406	0.068*	
C18	0.0106 (9)	−0.4122 (4)	0.1497 (2)	0.0809 (13)	
H18A	0.0434	−0.3882	0.1895	0.121*	
H18B	−0.0408	−0.4897	0.1502	0.121*	
H18C	−0.1035	−0.3641	0.1333	0.121*	

### Atomic displacement parameters (Å^2^)

**Table:**

	*U*^11^	*U*^22^	*U*^33^	*U*^12^	*U*^13^	*U*^23^
O1	0.094 (2)	0.0597 (15)	0.0483 (13)	0.0106 (16)	−0.0219 (15)	0.0002 (11)
O2	0.216 (5)	0.103 (2)	0.0688 (18)	0.026 (3)	−0.067 (3)	−0.0170 (18)
O3	0.071 (2)	0.0778 (19)	0.110 (2)	0.0202 (19)	−0.0248 (19)	−0.0096 (18)
O4	0.090 (2)	0.0495 (15)	0.0830 (18)	−0.0081 (17)	0.0002 (19)	−0.0120 (13)
C1	0.139 (5)	0.058 (2)	0.0377 (18)	0.008 (3)	−0.018 (3)	0.0036 (17)
C2	0.124 (4)	0.061 (2)	0.045 (2)	0.003 (3)	0.016 (3)	0.0031 (18)
C3	0.073 (3)	0.053 (2)	0.0527 (19)	0.003 (2)	0.010 (2)	−0.0003 (16)
C4	0.056 (2)	0.0419 (17)	0.0511 (18)	−0.0029 (18)	0.0013 (18)	0.0013 (14)
C5	0.067 (3)	0.069 (3)	0.092 (3)	0.020 (3)	−0.025 (3)	−0.019 (2)
C6	0.061 (4)	0.057 (3)	0.061 (3)	−0.004 (3)	−0.006 (3)	−0.005 (2)
C6'	0.077 (11)	0.058 (8)	0.057 (8)	−0.006 (9)	−0.015 (8)	0.006 (6)
C7	0.058 (2)	0.052 (2)	0.0456 (18)	−0.002 (2)	−0.0049 (17)	−0.0024 (15)
C8	0.072 (3)	0.0501 (19)	0.0554 (19)	0.009 (2)	−0.010 (2)	−0.0061 (16)
C9	0.065 (2)	0.0471 (17)	0.0381 (16)	−0.002 (2)	−0.0074 (18)	0.0046 (14)
C10	0.083 (5)	0.082 (4)	0.044 (3)	0.000 (4)	0.001 (3)	0.011 (3)
C11	0.097 (6)	0.071 (4)	0.071 (4)	0.009 (4)	−0.022 (4)	−0.019 (3)
C10'	0.063 (9)	0.055 (8)	0.069 (8)	−0.007 (8)	0.002 (8)	−0.008 (7)
C11'	0.084 (13)	0.074 (10)	0.061 (9)	0.006 (10)	−0.007 (9)	−0.016 (8)
C12	0.062 (2)	0.0481 (19)	0.0382 (16)	0.0058 (19)	−0.0006 (17)	−0.0007 (14)
C13	0.063 (2)	0.0440 (18)	0.0500 (18)	0.0108 (19)	0.0036 (19)	−0.0043 (15)
C14	0.059 (2)	0.053 (2)	0.0510 (18)	0.005 (2)	0.0000 (19)	−0.0069 (16)
C15	0.064 (2)	0.0478 (19)	0.0477 (17)	0.000 (2)	−0.0131 (19)	−0.0059 (15)
C16	0.072 (3)	0.050 (2)	0.057 (2)	0.013 (2)	−0.002 (2)	−0.0141 (17)
C17	0.064 (2)	0.057 (2)	0.0498 (19)	0.007 (2)	0.0061 (19)	−0.0057 (16)
C18	0.083 (3)	0.061 (2)	0.099 (3)	−0.014 (3)	−0.002 (3)	0.003 (2)

### Geometric parameters (Å, °)

**Table:**

O1—C1	1.387 (5)	C8—C9	1.479 (5)
O1—C9	1.388 (4)	C8—H8A	0.9700
O2—C1	1.197 (6)	C8—H8B	0.9700
O3—C5	1.213 (5)	C10—H10A	0.9600
O4—C15	1.376 (4)	C10—H10B	0.9600
O4—C18	1.404 (6)	C10—H10C	0.9600
C1—C2	1.467 (7)	C11—H11A	0.9600
C2—C3	1.531 (5)	C11—H11B	0.9600
C2—H2A	0.9700	C11—H11C	0.9600
C2—H2B	0.9700	C10'—H10D	0.9600
C3—C4	1.500 (5)	C10'—H10E	0.9600
C3—C12	1.510 (5)	C10'—H10F	0.9600
C3—H3	0.9800	C11'—H11D	0.9600
C4—C9	1.331 (5)	C11'—H11E	0.9600
C4—C5	1.455 (5)	C11'—H11F	0.9600
C5—C6	1.517 (6)	C12—C13	1.386 (5)
C5—C6'	1.592 (16)	C12—C17	1.395 (5)
C6—C7	1.539 (7)	C13—C14	1.383 (5)
C6—H6A	0.9700	C13—H13	0.9300
C6—H6B	0.9700	C14—C15	1.379 (5)
C6'—C7	1.532 (15)	C14—H14	0.9300
C6'—H6'1	0.9700	C15—C16	1.386 (6)
C6'—H6'2	0.9700	C16—C17	1.374 (5)
C7—C11	1.503 (7)	C16—H16	0.9300
C7—C8	1.528 (5)	C17—H17	0.9300
C7—C10	1.533 (6)	C18—H18A	0.9600
C7—C10'	1.542 (14)	C18—H18B	0.9600
C7—C11'	1.564 (16)	C18—H18C	0.9600
			
C1—O1—C9	119.0 (3)	C6'—C7—C11'	116.4 (9)
C15—O4—C18	117.6 (3)	C10—C7—C11'	68.8 (8)
O2—C1—O1	115.1 (5)	C6—C7—C11'	137.5 (8)
O2—C1—C2	127.8 (4)	C10'—C7—C11'	103.6 (10)
O1—C1—C2	117.1 (4)	C9—C8—C7	113.8 (3)
C1—C2—C3	112.0 (4)	C9—C8—H8A	108.8
C1—C2—H2A	109.2	C7—C8—H8A	108.8
C3—C2—H2A	109.2	C9—C8—H8B	108.8
C1—C2—H2B	109.2	C7—C8—H8B	108.8
C3—C2—H2B	109.2	H8A—C8—H8B	107.7
H2A—C2—H2B	107.9	C4—C9—O1	122.4 (3)
C4—C3—C12	114.6 (3)	C4—C9—C8	127.1 (3)
C4—C3—C2	106.9 (3)	O1—C9—C8	110.4 (3)
C12—C3—C2	111.4 (3)	C7—C10—H10A	109.5
C4—C3—H3	107.9	C7—C10—H10B	109.5
C12—C3—H3	107.9	C7—C10—H10C	109.5
C2—C3—H3	107.9	C7—C11—H11A	109.5
C9—C4—C5	118.8 (3)	C7—C11—H11B	109.5
C9—C4—C3	120.5 (3)	C7—C11—H11C	109.5
C5—C4—C3	120.6 (3)	C7—C10'—H10D	109.5
O3—C5—C4	123.0 (4)	C7—C10'—H10E	109.5
O3—C5—C6	120.3 (4)	H10D—C10'—H10E	109.5
C4—C5—C6	115.2 (4)	C7—C10'—H10F	109.5
O3—C5—C6'	114.5 (6)	H10D—C10'—H10F	109.5
C4—C5—C6'	110.5 (6)	H10E—C10'—H10F	109.5
C6—C5—C6'	49.1 (6)	C7—C11'—H11D	109.5
C5—C6—C7	112.0 (4)	C7—C11'—H11E	109.5
C5—C6—H6A	109.2	H11D—C11'—H11E	109.5
C7—C6—H6A	109.2	C7—C11'—H11F	109.5
C5—C6—H6B	109.2	H11D—C11'—H11F	109.5
C7—C6—H6B	109.2	H11E—C11'—H11F	109.5
H6A—C6—H6B	107.9	C13—C12—C17	117.3 (3)
C7—C6'—C5	108.4 (8)	C13—C12—C3	122.9 (3)
C7—C6'—H6'1	110.0	C17—C12—C3	119.7 (3)
C5—C6'—H6'1	110.0	C14—C13—C12	122.0 (3)
C7—C6'—H6'2	110.0	C14—C13—H13	119.0
C5—C6'—H6'2	110.0	C12—C13—H13	119.0
H6'1—C6'—H6'2	108.4	C15—C14—C13	119.5 (3)
C11—C7—C8	111.8 (4)	C15—C14—H14	120.3
C11—C7—C6'	132.9 (6)	C13—C14—H14	120.3
C8—C7—C6'	115.1 (6)	O4—C15—C14	125.0 (4)
C11—C7—C10	111.5 (5)	O4—C15—C16	115.4 (3)
C8—C7—C10	106.9 (4)	C14—C15—C16	119.6 (4)
C6'—C7—C10	58.7 (7)	C17—C16—C15	120.4 (3)
C11—C7—C6	109.7 (5)	C17—C16—H16	119.8
C8—C7—C6	108.8 (3)	C15—C16—H16	119.8
C6'—C7—C6	49.8 (7)	C16—C17—C12	121.2 (4)
C10—C7—C6	108.1 (4)	C16—C17—H17	119.4
C11—C7—C10'	59.3 (6)	C12—C17—H17	119.4
C8—C7—C10'	100.5 (6)	O4—C18—H18A	109.5
C6'—C7—C10'	106.5 (9)	O4—C18—H18B	109.5
C10—C7—C10'	152.4 (7)	H18A—C18—H18B	109.5
C6—C7—C10'	58.6 (6)	O4—C18—H18C	109.5
C11—C7—C11'	44.9 (7)	H18A—C18—H18C	109.5
C8—C7—C11'	112.5 (7)	H18B—C18—H18C	109.5
			
C9—O1—C1—O2	173.9 (4)	C5—C6—C7—C10'	−147.4 (8)
C9—O1—C1—C2	−6.4 (5)	C5—C6—C7—C11'	137.6 (12)
O2—C1—C2—C3	−138.4 (5)	C11—C7—C8—C9	158.7 (5)
O1—C1—C2—C3	41.9 (5)	C6'—C7—C8—C9	−16.1 (8)
C1—C2—C3—C4	−51.9 (5)	C10—C7—C8—C9	−79.0 (5)
C1—C2—C3—C12	73.9 (4)	C6—C7—C8—C9	37.5 (5)
C12—C3—C4—C9	−92.1 (4)	C10'—C7—C8—C9	97.7 (6)
C2—C3—C4—C9	31.8 (5)	C11'—C7—C8—C9	−152.6 (9)
C12—C3—C4—C5	90.9 (5)	C5—C4—C9—O1	179.4 (4)
C2—C3—C4—C5	−145.2 (4)	C3—C4—C9—O1	2.3 (5)
C9—C4—C5—O3	177.6 (4)	C5—C4—C9—C8	−3.2 (6)
C3—C4—C5—O3	−5.3 (7)	C3—C4—C9—C8	179.7 (4)
C9—C4—C5—C6	−16.1 (6)	C1—O1—C9—C4	−17.4 (5)
C3—C4—C5—C6	161.0 (4)	C1—O1—C9—C8	164.8 (3)
C9—C4—C5—C6'	37.3 (7)	C7—C8—C9—C4	−8.8 (5)
C3—C4—C5—C6'	−145.6 (6)	C7—C8—C9—O1	168.8 (3)
O3—C5—C6—C7	−146.6 (5)	C4—C3—C12—C13	28.7 (5)
C4—C5—C6—C7	46.7 (6)	C2—C3—C12—C13	−92.8 (4)
C6'—C5—C6—C7	−49.2 (8)	C4—C3—C12—C17	−154.6 (3)
O3—C5—C6'—C7	157.8 (7)	C2—C3—C12—C17	83.9 (4)
C4—C5—C6'—C7	−58.2 (10)	C17—C12—C13—C14	1.0 (5)
C6—C5—C6'—C7	48.0 (7)	C3—C12—C13—C14	177.8 (3)
C5—C6'—C7—C11	−125.9 (8)	C12—C13—C14—C15	−0.3 (5)
C5—C6'—C7—C8	47.5 (11)	C18—O4—C15—C14	−3.3 (5)
C5—C6'—C7—C10	142.8 (11)	C18—O4—C15—C16	176.7 (3)
C5—C6'—C7—C6	−46.5 (7)	C13—C14—C15—O4	179.5 (3)
C5—C6'—C7—C10'	−62.9 (11)	C13—C14—C15—C16	−0.5 (5)
C5—C6'—C7—C11'	−177.7 (10)	O4—C15—C16—C17	−179.4 (3)
C5—C6—C7—C11	−178.8 (4)	C14—C15—C16—C17	0.6 (5)
C5—C6—C7—C8	−56.3 (5)	C15—C16—C17—C12	0.1 (6)
C5—C6—C7—C6'	51.1 (7)	C13—C12—C17—C16	−0.9 (5)
C5—C6—C7—C10	59.4 (5)	C3—C12—C17—C16	−177.8 (4)
